# Expression and Roles of Teneurins in Zebrafish

**DOI:** 10.3389/fnins.2019.00158

**Published:** 2019-03-12

**Authors:** Angela Cheung, Katherine E. Trevers, Marta Reyes-Corral, Paride Antinucci, Robert Hindges

**Affiliations:** ^1^Centre for Developmental Neurobiology, King’s College London, London, United Kingdom; ^2^MRC Centre for Neurodevelopmental Disorders, King’s College London, London, United Kingdom

**Keywords:** teneurin/Odz, retinal ganglion cell, amacrine cell, visual system, synapse adhesion molecule, zebrafish

## Abstract

The teneurins, also known as Ten-m/Odz, are highly conserved type II transmembrane glycoproteins widely expressed throughout the nervous system. Functioning as dimers, these large cell-surface adhesion proteins play a key role in regulating neurodevelopmental processes such as axon targeting, synaptogenesis and neuronal wiring. Synaptic specificity is driven by molecular interactions, which can occur either in a trans-homophilic manner between teneurins or through a trans-heterophilic interaction across the synaptic cleft between teneurins and other cell-adhesion molecules, such as latrophilins. The significance of teneurins interactions during development is reflected in the widespread expression pattern of the four existing paralogs across interconnected regions of the nervous system, which we demonstrate here via *in situ* hybridization and the generation of transgenic BAC reporter lines in zebrafish. Focusing on the visual system, we will also highlight the recent developments that have been made in furthering our understanding of teneurin interactions and their functionality, including the instructive role of teneurin-3 in specifying the functional wiring of distinct amacrine and retinal ganglion cells in the vertebrate visual system underlying a particular functionality. Based on the distinct expression pattern of all teneurins in different retinal cells, it is conceivable that the combination of different teneurins is crucial for the generation of discrete visual circuits. Finally, mutations in all four human teneurin genes have been linked to several types of neurodevelopmental disorders. The opportunity therefore arises that findings about the roles of zebrafish teneurins or their orthologs in other species shed light on the molecular mechanisms in the etiology of such human disorders.

## Introduction

As one of the most complex systems in nature, the functionality of the nervous system is highly dependent on the formation of precise synaptic connections between neurons during development. While progress is still being made in furthering our understanding of these mechanisms, it is becoming increasingly evident that synaptic specificity is a finely attuned process involving a plethora of cell adhesion molecules that act in a combinatorial manner to generate diverse cellular interactions. The teneurins, also known as Ten-m/Odz, are one family of such cell adhesion molecules that has been implicated, among others, in regulating the specificity of synaptic connections.

A phylogenetically conserved family of type II transmembrane glycoproteins first discovered in the early 1990s in *Drosophila*, the teneurins have been shown to be involved in intercellular signaling during development (Tucker and Chiquet-Ehrismann, 2006; Tucker et al., 2012). Their key role in mediating basic neurodevelopmental processes such as axon guidance and synaptic partner matching (Drabikowski et al., 2005; Hong et al., 2012) is reflected in the high expression of teneurins in the central nervous system. Across species, *C. elegans* has a single teneurin (Ten-1), *Drosophila* have two (Ten-m and Ten-a) and all vertebrates have four paralogs (tenm1–4). Sequence similarity is high between paralogs, with human teneurin paralogs sharing 58–70% sequence identity (Jackson et al., 2018).

**FIGURE 1:**
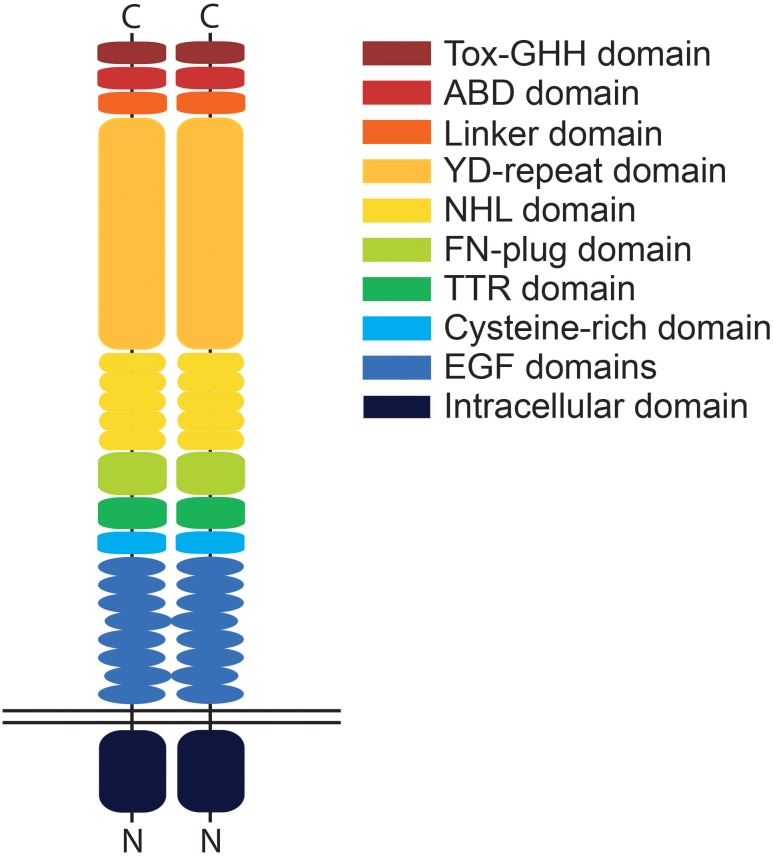
Schematic illustration of a teneurin dimer. Teneurins possess a single transmembrane domain and small intracellular domain compared to the relatively large extracellular domain. Depicted is a *cis*-dimer with identified protein domains indicated in different colors. The extracellular domain consists of the EGF domain, cysteine-rich domain, TTR (transthyretin-related) domain, FN (fibronectin)-plug domain, NHL domain, YD (tyrosine-aspartate)-repeat domain, internal linker domain, ABD (antibiotic-binding domain-like) domain and the Tox-GHH domain. The EGF domains play a key role in regulating *cis*-interactions between teneurin molecules while the NHL- and YD-repeat domains mediate trans-interactions. Domains are only representative and not to relative scale.

The teneurins themselves are large proteins of around 300 kDa with a smaller N-terminal intracellular domain, a single span transmembrane domain and large C-terminal extracellular region (Rubin et al., 1999; Tucker and Chiquet-Ehrismann, 2006; Figure 1). While the intracellular domain is able to interact with the cytoskeleton, as has been shown with tenm1 (Nunes et al., 2005), the highly conserved 200 kDa extracellular domain of teneurin, which contains eight epidermal growth factor (EGF)-like repeats and five NHL (NCL-1, HT2A, and Lin-41) repeats, dimerizes in *cis* to mediate both homo- and heterophilic interactions (Beckmann et al., 2013). In addition, the teneurins can interact trans-synaptically with other cell adhesion molecules such as latrophilins via their NHL domains (Boucard et al., 2014). Tenm2, for example, interacts across the synaptic cleft with presynaptic latrophilin1 to mediate calcium signaling and synapse formation (Vysokov et al., 2016). Recent X-ray crystallography and cryo-EM imaging data has demonstrated that the ectodomains of tenm2 and 3 exist in a large β-barrel conformation consisting of an eight sub-domain super-fold structured on a spiraling YD-repeat shell domain (Jackson et al., 2018; Li et al., 2018). Further elucidation of the structural interactions between teneurins and other proteins would provide insight into functionality and factors driving the molecular diversity underpinning synaptic connectivity or even highlight possible new interaction partners.

Indeed, the importance of teneurins in regulating synaptic partner matching and functional connectivity is well demonstrated in the vertebrate visual system where retinal ganglion cells (RGCs) form specific connections with their synaptic partners. In zebrafish, tenm3 is required by RGCs and amacrine cells for acquiring correct structural and functional connectivity *in vivo*, with tenm3 knockdown or knockout leading to defects in RGC and amacrine cell dendrite stratification in the retina and the disrupted development of orientation selectivity (Antinucci et al., 2013, 2016). Tenm3 has also recently been shown to regulate topographic circuit assembly in the hippocampus of mice (Berns et al., 2018). More broadly, the teneurins are strongly implicated in the establishment of visual mapping in mice (Leamey and Sawatari, 2014). Anterograde tracing of RGC axons in tenm2 and tenm3 knockout mice showed notable aberrant changes in the mapping of ipsilateral projections from the retina to the dorsal lateral geniculate nucleus (dLGN) and the superior colliculus (Leamey et al., 2007; Young et al., 2013).

Although the fundamental role of teneurins in establishing synaptic connectivity is becoming increasingly apparent, data on the spatio-temporal expression pattern of teneurins across the central nervous system, and its functional and physiological significance, is largely lacking. Through *in situ* hybridization and the generation of transgenic bacterial artificial chromosome (BAC) reporter lines in zebrafish, we present the expression patterns of teneurin 1–4 across the central nervous system and identify some of the teneurin-positive cell types, focusing particularly on the visual system.

## Materials and Methods

### Zebrafish Husbandry

Zebrafish (*Danio rerio*) adults and embryos were maintained in accordance with the Animals (Scientific Procedures) Act 1986 under license from the United Kingdom Home Office (PPL70/9036).

Pairwise zebrafish spawnings were set up using Ekkwill wild type adults (Ekkwill Breeders, Florida). Larvae were maintained between 0 and 5 days post fertilization (dpf) at 28.5°C on a 14 h ON/10 h OFF light cycle in 1 × Danieau solution (58 mM NaCl, 0.7 mM KCl, 0.6 mM Ca(NO_3_)_2_, 0.4 mM MgSO_4_, 5 mM HEPES) supplemented with PTU (Sigma) at a final concentration of 200 μM. Where necessary, chorions were removed using forceps. Larvae were anesthetized with MS222 and fixed overnight with 4% paraformaldehyde (PFA) in PBS at 4°C, then dehydrated in 100% methanol and stored at -20°C.

### RNA Probe Synthesis

cDNAs spanning several exons of each tenm were blunt cloned into the pSC-B-Amp plasmid using the following primers: tenm1 exons 19–22 Fwd: gatctcagcaggaatgtggagg, Rev: cagcatcccggcgttactgatg; tenm2 exons 27–29 Fwd: gcatgtgttcaaccactcca, Rev: gcgccatttaacaccagaac; tenm3 exons 26–28 Fwd: gggactatgacattcaagcaggtc, Rev: cattgttggcactgtcggccag; tenm4 exons 21–25 Fwd: catttcccagcagcctccagtc, Rev: ctttcgcgtagccgtcgtcg. Plasmids were linearized and transcribed using NotI and T3 (for tenm1, tenm3, tenm4) or EcoRV and T7 (for tenm2) to generate DIG-labeled anti-sense riboprobes. Probes were purified by LiCl precipitation and suspended in ultrapure water. Working probes were diluted to 250 ng/ml in hybridization mix (HM) containing 50% formamide, 5 × SSC pH 6.0, 0.1% Tween-20, 50 μg/ml Heparin and 500 μg/ml tRNA.

### Wholemount *in situ* Hybridization

*In situ* hybridization was performed according to Thisse and Thisse (2008). In brief, samples were rehydrated through a series of 75, 50, and 25% methanol, into PBS containing 0.1% Tween-20 (PBTw) and permeabilized by digestion with 10 μg/ml Proteinase K in PBTw at room temperature. Digestion times varied according to developmental stage (10 min for 1 dpf, 20 min for 2 dpf and 30 min for 3–5 dpf). Digestion was stopped by post-fixing the embryos in 4% PFA in PBS for 20 min at room temperature. Residual fix was removed by washing 2 × 10 min in PBTw before embryos were prehybridized in HM at 70°C for 3 h. Probes were hybridized overnight at 70°C and the embryos were subsequently washed 1 × 10 min through 75, 50, and 25% HM, into 2 × SSC followed by 2 × 30 min washes with 0.2 × SSC, all at 70°C. Then, at room temperature, the larvae were washed through a series of 75, 50, and 25% 0.2 × SSC, into PBTw before non-specific binding was blocked for 3 h at room temperature using 2 mg/ml BSA and 2% sheep serum in PBTw. Samples were incubated overnight at 4°C in blocking buffer containing anti-DIG antibody (Roche) diluted 1:10,000. Excess antibody was removed by washing 4 × 1 h in PBTw. Finally, samples were washed 2 × 15 min in staining buffer containing 10 mM Tris–HCl pH 9.5, 100 mM NaCl and 0.1% Tween-20 and color staining was developed in the dark at room temperature using NBT/BCIP stock solution (Roche) diluted in staining buffer. The color reaction was checked frequently and stopped by washing in PBTw. Images were captured using a Leica M165 FC stereomicroscope, QImaging Retiga camera and Volocity software.

### Paraffin Sections

Larvae were post-fixed overnight in 4% PFA at room temperature and dehydrated for 30 min in 100% methanol and 30 min in isopropanol. Samples were cleared with tetrahydronaphthalene (THN) for 30 min. Molten Paraplast wax (Sigma) was added to give a 1:1 mix of THN:wax and incubated at 60°C for 30 min. This was replaced 3 × 1 h with fresh molten wax at 60°C before samples were oriented, embedded and left to solidify. 12 μm transverse sections were cut on a Microm HM315 microtome and mounted on slides treated with glycerin albumin. Slides were dried overnight at 37°C and dewaxed by 2 × 4 h washes in Histoclear II. Coverslips were mounted using a 1:3 mixture of Histoclear II: Canada Balsam. Images were captured using an Olympus Vanox-T with 40 objectives and a QImaging Retiga 2000R camera with QCapture Pro software.

### BAC Transgenesis

The BAC clones containing the gene sequences for tenm 1, 2.14 and 4 were identified by using the ENSEMBL database of the Wellcome Trust Sanger Institute (Daniokey library reference: tenm1, DKEY-275B22; tenm2.14, DKEY-47H20; tenm4, DKEY-84I3). Zebrafish have two genes that encode for tenm2, tenm2.14 and tenm2.21, present on chromosomes 14 and 21, respectively (Supplementary Figure S1). The tenm2.14 paralog was chosen for further investigation, based on its higher resemblance to other tenm2 orthologs. BAC clones were transformed first with a pRed-Flp4 plasmid before a recombineering step was used to insert a membrane-localized reporter gene (mCitrine) together with a kanamycin resistance cassette at the beginning of the tenm gene. Transient transgenic zebrafish lines were created by microinjection of teneurin:mCitrine BAC constructs into one-cell stage embryos, before embryos were allowed to develop at 28.5°C with PTU until 3–5 dpf. Embryos were mounted laterally on glass slides using 1% low-melting point agarose in Danieau’s solution and imaged on an LSM 710 Zeiss confocal equipped with a spectral detection scan head and a 20×/1.0 NA water-immersion objective.

## Results

### Tenm1–4 Are Widely Expressed Across the Central Nervous System

**FIGURE 2:**
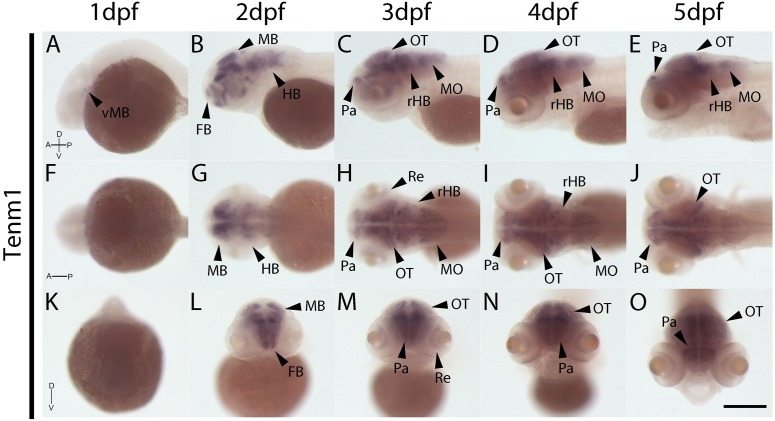
Wholemount expression of tenm1 during zebrafish development. Tenm1 expression during 1–5 dpf shown from lateral **(A–E)**, dorsal **(F–J)** and frontal **(K–O)** perspectives. A, Anterior; D, Dorsal; FB, Forebrain; HB, Hindbrain; MB, Midbrain; MO, Medulla Oblongata; OT, Optic Tectum; Pa, Pallium; P, Posterior; Re, Retina; rHB, rostral Hindbrain; V, Ventral; vMB, ventral Midbrain. Scale bar in all panels = 250 μm.

**FIGURE 3:**
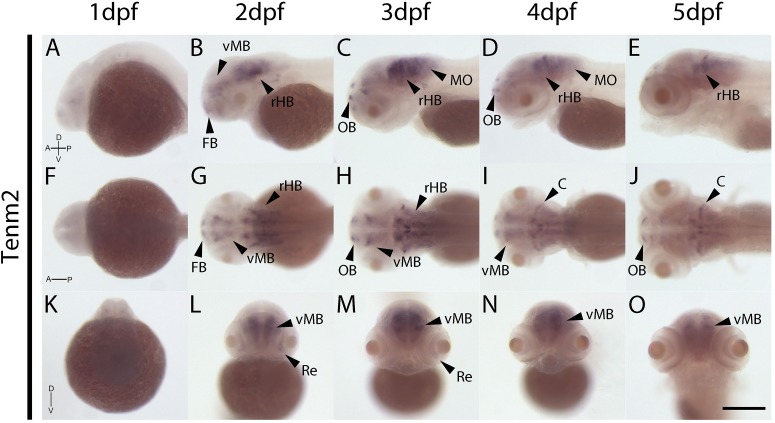
Wholemount expression of tenm2 during zebrafish development. Tenm2 expression during 1–5 dpf shown from lateral **(A–E)**, dorsal **(F–J)** and frontal **(K–O)** perspectives. A, Anterior; C, Cerebellum; D, Dorsal; FB, Forebrain; MO, Medulla Oblongata; OB, Olfactory Bulb; P, Posterior; Re, Retina; rHB, rostral Hindbrain; V, Ventral; vMB, ventral Midbrain. Scale bar in all panels = 250 μm.

The expression patterns of the four vertebrate paralogs of teneurin were investigated via wholemount *in situ* hybridization on zebrafish embryos over 1 to 5 dpf. Tenm1 is first detected in a discrete spot in the ventral midbrain at 1 dpf (Figure 2A; arrow). Later expression is more widespread but localized anteriorly with tenm1 observed in multiple regions of the forebrain, midbrain and hindbrain at 2–5 dpf (Figure 2). Similarly, significant tenm2 expression is first detected anteriorly at 2 dpf in the olfactory bulbs and in multiple clusters of neurons in the midbrain and hindbrain, persisting over 3–5 dpf (Figure 3).

**FIGURE 4:**
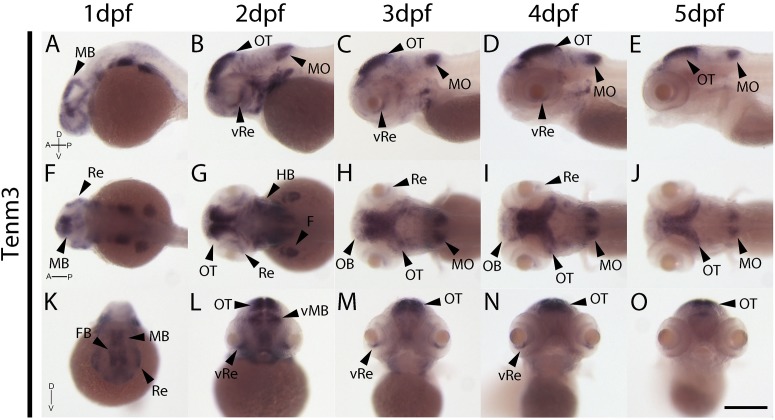
Wholemount expression of tenm3 during zebrafish development. Tenm3 expression during 1–5 dpf from lateral **(A–E)**, dorsal **(F–J)** and frontal **(K–O)** perspectives. A, Anterior; D, Dorsal; F, Fin; FB, Forebrain; HB, Hindbrain; MB, Midbrain; MO, Medulla Oblongata; OB, Olfactory Bulb; OT, Optic Tectum; P, Posterior; Re, Retina; V, Ventral; vRe, ventral Retina. Scale bar in all panels = 250 μm.

**FIGURE 5:**
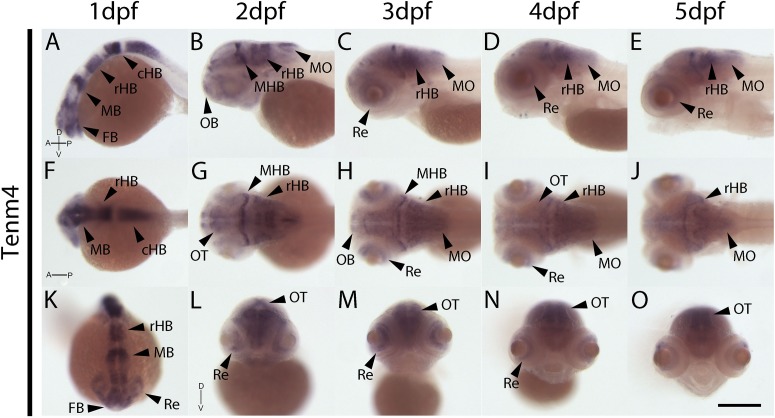
Wholemount expression of tenm4 during zebrafish development. Tenm4 expression during 1–5 dpf from lateral **(A–E)**, dorsal **(F–J)** and frontal **(K–O)** perspectives. A, Anterior; cHB, caudal Hindbrain; D, Dorsal; FB, Forebrain; MB, Midbrain; MHB, Mid-Hindbrain Boundary; MO, Medulla Oblongata; OB, Olfactory Bulb; OT, Optic Tectum; P, Posterior; rHB, rostral Hindbrain; Re, Retina; V, Ventral. Scale bar in all panels = 250 μm.

In contrast to tenm1 and 2, which were either weak or absent at 1 dpf, strong tenm3 expression is already detected early at 1 dpf in the forebrain and midbrain, as well as the developing retina (Figure 4A,F,K). By 2 dpf, high expression is specifically localized to the optic tectum, ventral retina, medulla oblongata and tips of the fin buds, persisting over 3–5 dpf (Figure 4). Similarly, strong tenm4 expression is also detected early at 1 dpf in the forming retina, midbrain and hindbrain, and becomes localized to the inner layers of the retina, olfactory bulbs, optic tectum, subset of hindbrain neurons, and along the mid-hindbrain boundary at 2–3 dpf. This expression persists over 4–5 dpf (Figure 5).

### The Teneurins Are Expressed Across Interconnected Regions of the Zebrafish Nervous System During Development

Transverse sections across the zebrafish central nervous system were collected from wholemount *in situ* hybridization samples in order to gain more detailed insight into the expression pattern of teneurins from 1 to 4 dpf.

**FIGURE 6:**
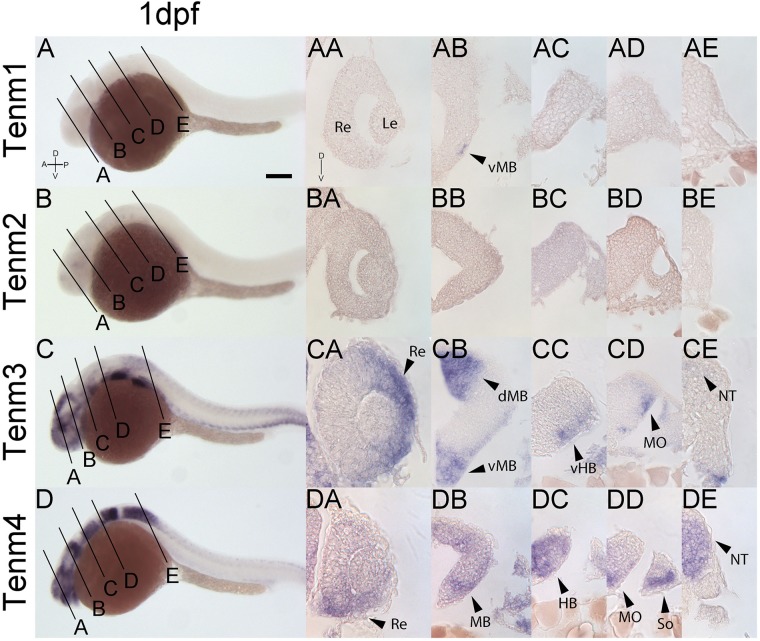
Comparing tenm1–4 expression at 1 dpf. Transverse sections through the retina **(A)**, midbrain **(B)**, rostral hindbrain **(C)**, caudal hindbrain **(D)** and spinal cord **(E)** of 1 dpf zebrafish expressing tenm1–4. A, Anterior; D, Dorsal; dMB, dorsal Midbrain; HB, Hindbrain; Le, Lens; MB, Midbrain; MO, Medulla Oblongata; NT, Neural Tube; P, Posterior; Re, Retina; So, Somites; vMB, ventral Midbrain; V, Ventral; vHB, ventral Hindbrain. Scale bar = 150 μm in **A–D**; 30 μm in all other panels.

At 1 dpf, tenm1 is only expressed in a discrete population of cells in the ventral midbrain (Figure 6AB; arrow), while tenm2 expression is absent. In contrast, tenm3 and tenm4 are expressed strongly in broad domains of the forming retina and midbrain (Figure 6CA, DA, CB, DB). Tenm4 is expressed throughout the rostral and caudal hindbrain (Figure 6DC, DD), whereas tenm3 is restricted laterally (Figure 6CC, CD). While tenm4 is strongly expressed in the neural tube (Figure 6DE), tenm3 expression is much weaker (Figure 6CE).

**FIGURE 7:**
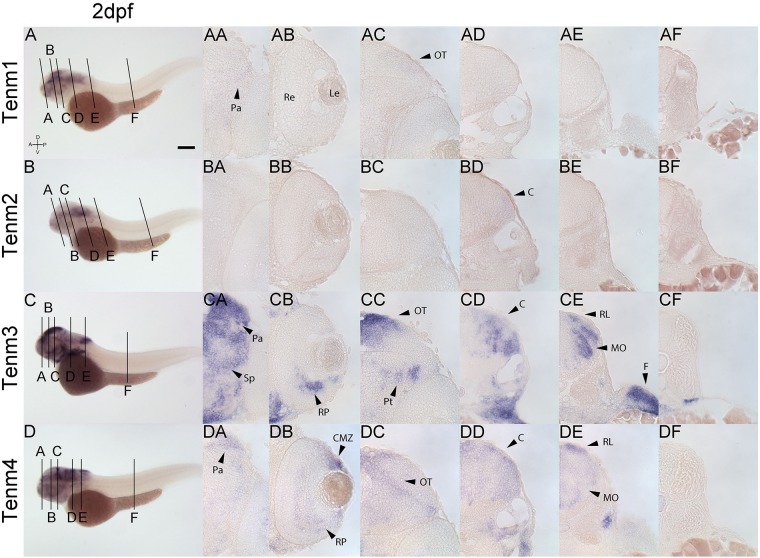
Comparing tenm1–4 expression at 2 dpf. Transverse sections through the forebrain **(A)**, retina **(B)**, midbrain **(C)**, rostral hindbrain **(D)**, caudal hindbrain **(E)** and spinal cord **(F)** of 2 dpf zebrafish expressing tenm1–4. A, Anterior; C, Cerebellum; CMZ, Ciliary Marginal Zone; D, Dorsal; F, Fin; Le, lens; MO, Medulla Oblongata; OT, Optic Tectum; Pa, Pallium; P, Posterior; Pt, Pretectum; Re, Retina; RL, Rhombic Lip; Rp, Retinal Progenitors; Sp, Subpallium; V, Ventral. Scale bar = 250 μm in **A–D**; 30 μm in all other panels.

By 2 dpf, tenm3 and tenm4 are expressed strongly in the forebrain pallium (Figure 7CA, DA), while tenm1 transcripts are only faintly detected (Figure 7AA). In the retina, tenm3 is restricted to retinal progenitors in the inner ventral domain (Figure 7CB), while tenm4 is expressed more broadly across the retina and at the ciliary marginal zone (Figure 7DB). Neither tenm1 nor tenm2 are detected in the retina at these stages (Figure 7AB, BB). Tectal cells express tenm4 broadly (Figure 7DC), whereas tenm3 is restricted dorsally (Figure 7CC). A weak expression of tenm1 can also be observed in the lateral tectum (Figure 7AC), and overlapping tenm2, 3 and 4 expression (Figure 7BD, CD, DD) is detected in the cerebellum of the rostral hindbrain, but only tenm3 and tenm4 are observed at the rhombic lip and medulla oblongata of the caudal hindbrain (Figure 7CE, DE). No teneurin expression is detected in the spinal cord at 2 dpf.

**FIGURE 8:**
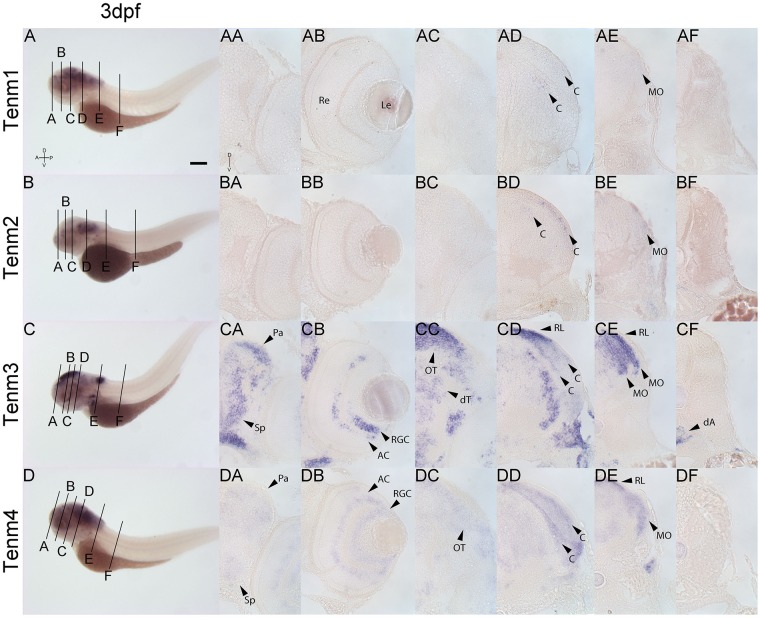
Comparing tenm1–4 expression at 3 dpf. Transverse sections through the forebrain **(A)**, retina **(B)**, midbrain **(C)**, rostral hindbrain **(D)**, caudal hindbrain **(E)** and spinal cord **(F)** of 3 dpf zebrafish expressing tenm1–4. A, Anterior; AC, Amacrine Cells; C, Cerebellum; dA, dorsal Aorta; D, Dorsal; dT, dorsal Thalamus; Le, Lens; MO, Medulla Oblongata; OT, Optic Tectum; Pa, Pallium; P, Posterior; Re, Retina; RGC, Retinal Ganglion Cells; RL, Rhombic Lip; Sp, Subpallium; V, Ventral. Scale bar = 250 μm in **A–D**; 30 μm in all other panels.

Tenm1 and tenm2 are not detected in the forebrain, retina or midbrain at 3 dpf. Tenm3 and tenm4 can be detected in the pallium and subpallium, but the latter only weakly (Figure 8CA, DA). Tenm3 and tenm4 are also detected in amacrine cells, retinal ganglion cells (Figure 8CB, DB) and in tectal neurons (Figure 8CC, DC). All teneurins are expressed in the rostral and caudal hindbrain (Figure 8AD, AE, BD, BE, CD, CE, DD, DE) but in different layers of the cerebellum and medulla oblongata, with tenm1 and tenm2 expression weak in this area. Tenm3 and tenm4 are also present at the rhombic lip, while again, no teneurin expression is observed along the spinal cord.

**FIGURE 9:**
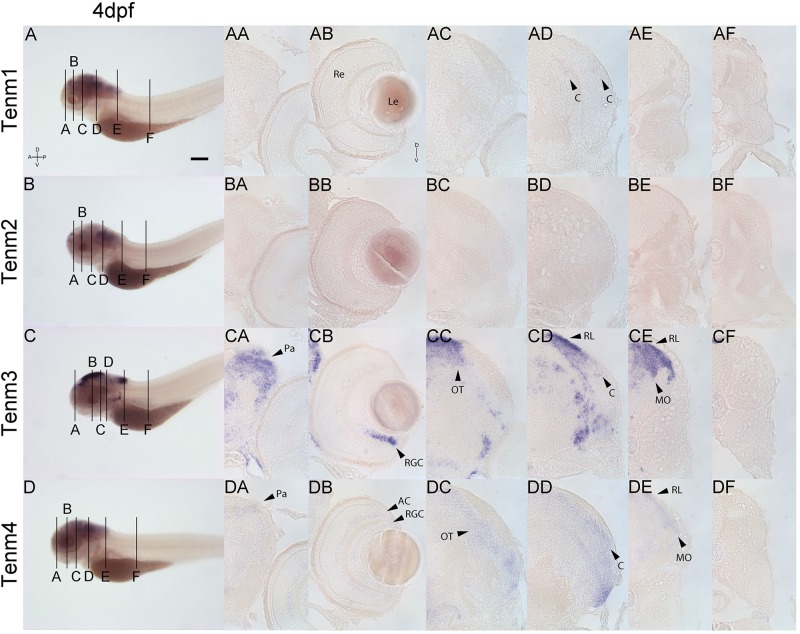
Comparing tenm1–4 expression at 4 dpf. Transverse sections through the forebrain **(A)**, retina **(B)**, midbrain **(C)**, rostral hindbrain **(D)**, caudal hindbrain **(E)** and spinal cord **(F)** of 4 dpf zebrafish expressing tenm1–4. A, Anterior; AC, Amacrine Cells; C, Cerebellum; D, Dorsal; Le, Lens; MO, Medulla Oblongata; OT, Optic Tectum; Pa, Pallium; P, Posterior; Re, Retina; RGC, Retinal Ganglion Cells; RL, Rhombic Lip; V, Ventral. Scale bar = 250 μm in **A–D**; 30 μm in all other panels.

Finally, at 4 dpf, tenm3 and tenm4 expression persists in the pallium (Figure 9CA, DA), while in the retina, tenm3 mRNA is no longer detected in amacrine cells and remains only in the ventral population of RGCs (Figure 9CB). Tenm4 expression in these cells is also reduced compared to 3 dpf (Figure 9DB), while both tenm3 and tenm4 are present in tectal cells (Figure 9CC, DC), as well as in the cerebellum, rhombic lip and medulla oblongata (Figure 9CD, CE, DD, DE). Very weak tenm1 expression overlaps in the cerebellum (Figure 9AD). Tenm2 expression is too weak to detect in sections at 4 dpf.

Although teneurin 1–4 were found to be widely expressed in overlapping regions of the nervous system, we cannot infer from our studies whether different teneurin paralogs are co-expressed within individual cells.

### The Teneurins Are Expressed in MultipleCell Types of the Retina

To further investigate the types of retinal cells that are positive for individual teneurins, we generated BAC constructs for tenm1, 2.14 and 4, inserting the coding sequence for the fluorescent reporter mCitrine at the place of the ATG in the genomic locus (Supplementary Figure S1).

**FIGURE 10:**
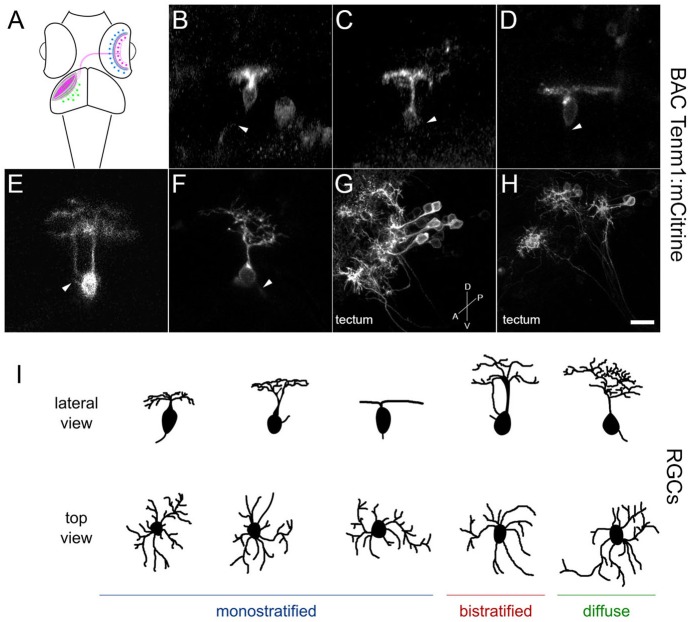
Tenm1-positive cells in the zebrafish visual system in tenm1:mCitrine transient transgenic fish at 3 dpf. Schematic of amacrine cells (blue), RGCs (magenta) and tectal cells (green) in the zebrafish visual system **(A)**. Lateral view of representative RGCs with monostratified **(B–D)**, bistratified **(E)** and diffuse **(F)** dendritic arbors. RGC axons are marked by arrowheads. Tenm1-positive tectal cells **(G–H)**. All images represent maximum intensity projections of ∼30 μm confocal z-stacks. Scale bar 10 μm **(B–F)**, 30 μm **(G)** and 20 μm **(H)**. Schematic representation of tenm1-positive RGC morphology based on representative RGCs imaged in tenm1:mCitrine transient transgenic fish at 3 dpf **(I)**.

We have previously described the expression of tenm3 in the zebrafish visual system in detail, including the identification of tenm3-positive retinal cells using a BAC transgenic strategy (Antinucci et al., 2013, 2016). Here we extend these analyses to describe retinal and tectal cells in zebrafish positive for other members of the teneurin family. Transient transgenic zebrafish embryos expressing teneurin:mCitrine BAC constructs were imaged at 3–5 dpf to investigate the morphology and distribution of tenm1-, tenm2.14- and tenm4-positive cells in the developing visual system, where teneurins have been shown to play a key role in establishing functional neural circuitry. All three BAC constructs tested labeled single cells of the visual system, including RGCs, amacrine cells and tectal cells (Figure 10A).

The tenm1:mCitrine BAC construct consistently labeled RGCs (Figure 10B–F) and a low number of amacrine cells in the retina, as well as cells in the optic tectum. All tenm1-positive tectal cells shared a characteristic elongated morphology with the axon projecting from the dendritic arbor instead of from the cell soma (Figure 10G,H). Citrine-expressing RGCs in the retina were classified morphologically into three main groups according to their arborization patterns (Figure 10I); monostratified, bistratified or diffuse. Monostratified RGCs were further subdivided into ON, OFF and asymmetric dendritic arbors according to the distance of the dendritic field to the cell body. Although all RGC types had similar dendritic field diameters, the monostratified asymmetric and diffuse asymmetric RGCs had the largest dendritic field coverage/area (Table 1).

**Table 1:** Mean dendritic field diameter and area of mCitrine-labeled retinal ganglion cells in tenm1-BAC-injected fish.

RGC morphology	Monostratified (ON)	Monostratified (OFF)	Monostratified asymmetric (ON)	Bistratified	Diffuse asymmetric
Dendritic field diameter (μm)	29 ± 2	27 ± 4	33	31	38 ± 5
Dendritic field area (μm^2^)	580 ± 20	600 ± 200	780	720	800 ± 200
*n*	2	3	1	1	2


*The dendritic field diameter was measured as the width of the dendritic arbor, while the dendritic field area was measured from the top views of the RGCs. Measurements were made with ImageJ. *n* represents the number of cells analyzed per type. SD is shown for all cell types where *n* > 1.*

**FIGURE 11:**
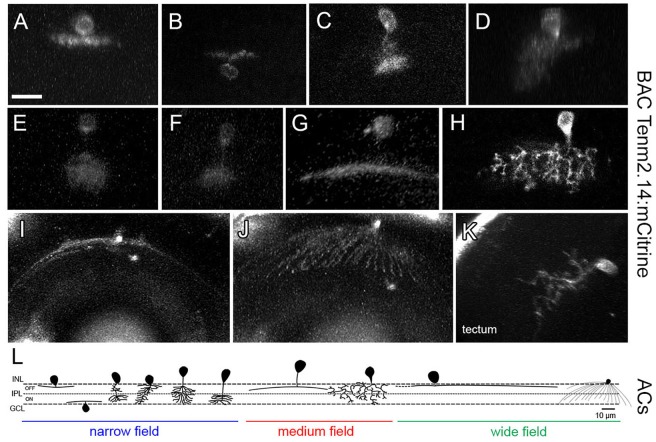
Tenm2.14-positive cells in the zebrafish visual system in tenm2.14:mCitrine transient transgenic fish at 4 and 5 dpf. Lateral view of representative narrow field **(A–F)**, medium field **(G–H)** and wide field **(I)** amacrine cells. Top view of a wide field amacrine cell showing ample dendritic coverage of the retina **(J)**. Lateral view of a tectal cell **(K)**. All images represent maximum intensity projections of ∼30 μm confocal z-stacks. Scale bar 10 μm **(A–H)**, 20 μm **(I–J)** and 15 μm **(K)**. Schematic representation of tenm2.14-positive amacrine cell morphology based on representative cells imaged in tenm2.14:mCitrine transient transgenic fish at 4 and 5 dpf **(L)**.

In direct contrast to tenm1:mCitrine, the tenm2.14:mCitrine BAC construct labeled predominantly amacrine cells (Figure 11A–J) along with a very low number of RGCs. Tectal cells were also labeled (Figure 11K), and similar to when the tenm1:mCitrine construct was used, the number of labeled cells was higher in the optic tectum than in the retina. For Tenm2.14:mCitrine, the fluorescently labeled cells could be classified into narrow-field, medium-field and wide-field amacrine cells according to their dendritic arborization and morphology, with further subdivision in accordance to their stratification within the inner plexiform layer (IPL); ON or OFF (Figure 11L). A total number of 9 amacrine cell types were defined: 6 narrow-field (2 monostratified and 4 diffuse), 2 medium-field (1 monostratified and 1 diffuse) and one wide-field (monostratified), with the narrow-field amacrine cells being the most abundant. The average dendritic field for narrow-field amacrine cells was 4 times and 14 times smaller than the average dendritic field of medium-field amacrine cells and wide-field amacrine cells, respectively (Table 2).

**Table 2:** Mean dendritic field diameter and area of mCitrine-labeled amacrine cells in tenm2.14-BAC-injected fish.

Amacrine cell morphology	Narrow field						Medium field		Wide field
			
	Monostratified		Diffuse				Monostratified	Diffuse	Monostratified
				
	OFF	ON	2 bunches	Asym.	ON/OFF	ON	OFF	Asym.	ON
Dendritic field diameter (μm)	22 ± 2	23 ± 3	13	24	16	12 ± 1	100 ± 30	50	230
Dendritic field area (μm^2^)	350 ± 70	310 ± 50	200	490	230	160 ± 20	1300 ± 300	1110	4080
*n*	8	5	1	1	1	2	2	1	1


*The dendritic field diameter was measured as the width of the dendritic arbor, while the dendritic field area was measured from the top views of the amacrine cells. Measurements were made with ImageJ. *n* represents the number of cells analyzed per type. SD is shown for all cell types where *n* > 1.*

While the tenm1:mCitrine and tenm2.14:mCitrine BAC constructs strongly labeled a range of distinct cell types, the tenm4:mCitrine BAC only lead to weak reporter expression in cells and further detailed morphological analyses could not be carried out.

## Discussion

We describe here the expression pattern of different members of the teneurin family across the central nervous system in zebrafish during development, focusing particularly on the visual system where they have been shown to play a pivotal role in establishing connectivity, via *in situ* hybridization and BAC transgenesis.

Although the teneurins are also expressed in non-neuronal tissues, such as at sites of cell migration and at muscle attachment points, they are primarily concentrated in the central nervous system and at sites of pattern formation during development (Baumgartner and Chiquet-Ehrismann, 1993; Baumgartner et al., 1994; Fascetti and Baumgartner, 2002; Drabikowski et al., 2005). This localization is observed across all teneurin expressing species and supports the role of teneurins in neurodevelopmental processes such as neurite outgrowth, axon guidance and synapse formation (Tucker and Chiquet-Ehrismann, 2006; Leamey and Sawatari, 2014). Consistent with that, we observed an early widespread expression of teneurins across the central nervous system with tenm1, 3 and 4 expressions already present in embryos at 1 dpf. Indeed, the early strong expression of tenm3 and 4 in the developing retina supports the role of teneurins, particularly of tenm3, in directing the functional connectivity of RGCs and amacrine cells in the developing visual system (Antinucci et al., 2013). Both genes remain strongly expressed in various structures of the developing central nervous system well into 4 dpf. Tenm1 and 2 expression, on the other hand, is not observed at all in the retina during development and overall expression is weak, especially by 4 dpf.

All four teneurins are also expressed at various levels in different layers of the cerebellum at 3 dpf. Interestingly, mutations in tenm3 have been implicated as a plausible candidate for a new dominantly inherited form of pure adult-onset cerebellar ataxia in humans (Storey et al., 2009). Essentially, all four teneurin paralogs are expressed in an overlapping manner across interconnected areas of the central nervous system of the zebrafish embryo, such as the retina and the optic tectum. The strong co-expression of tenm3 and 4 across many areas may be suggestive of a possible functional redundancy between the two teneurins, although this is still to be further investigated. Mieda et al. (1999), also performed *in situ* hybridization studies on tenm3 and 4 in the developing zebrafish embryo and showed that the expression patterns were complementary to each other early on in the developing embryo (<26 hpf) but becomes ambiguous at later stages.

While we are one of the first to present an in-depth study of the expression pattern of all four teneurin paralogs across the zebrafish embryo during development, comprehensive expression data is already available for other species such as the mouse. For example, Zhou et al. (2003) showed that the expression of the four teneurin genes in the developing and adult central nervous system of mice was distinct but partially overlapping. Similar to what we found in the zebrafish, the teneurins were expressed widely across the central nervous system and significant levels of tenm3 and 4 were detected early on during development (Zhou et al., 2003). All four teneurins show a graded expression across the cortex and striatum during embryonic and early postnatal stages (Li et al., 2006; Bibollet-Bahena et al., 2017), and intriguingly, tenm4 is also expressed in the subventricular zone, suggesting a possible role in determining cortical progenitor cell fate (Li et al., 2006).

While tenm3 is expressed in interconnected areas of the zebrafish visual system such as the retina and optic tectum, similarly, in the mouse visual system, tenm3 is expressed across interconnected regions of the retina, dLGN, superior colliculus (SC) and visual cortex (Leamey et al., 2007, 2008). Tenm2 and tenm4 in mice have also been shown to be expressed strongly in the interconnected visual cortex and dLGN (Li et al., 2006). Mouse hippocampal tenm expression also matches the topographic connectivity between the entorhinal cortex, CA1 and subiculum (Berns et al., 2018).

Zhou et al. (2003) also noted a differential distribution of mRNA transcripts and protein, which has also been previously observed in *Drosophila*, and which they explain as possible axonal transport into target regions (Baumgartner et al., 1994; Zhou et al., 2003). It would be insightful to investigate this further once reliable antibodies for detecting teneurin protein expression in zebrafish are available.

In the developing chick visual system, tenm1 and 2 have been found to be predominantly expressed in non-overlapping populations of neurons during the time of axonal growth in embryos (Rubin et al., 1999). Tenm1 is expressed in the IPL, the ganglion cell layer (GCL) and the tectum and displays a complementary pattern of expression with tenm2 in different sublaminae of the IPL in chick (Kenzelmann et al., 2008). To further investigate teneurin expression in the developing visual system of zebrafish, different teneurin:mCitrine BAC constructs were injected into zebrafish embryos in an attempt to create transient transgenic lines expressing tenm1, 2.14 and 4. Although tenm1 and 2 transcript expression was not observed in the retina with *in situ* hybridization techniques, BAC transgenesis enabled a more sensitive detection of lower levels of teneurin expression.

In addition to validating these BAC constructs, which consistently labeled both amacrine cells and RGCs, a preliminary classification of cells expressing teneurins in the inner retina and optic tectum was accomplished. While the tenm1:mCitrine BAC construct primarily labeled five subtypes of RGCs, the tenm2.14:mCitrine BAC labeled nine amacrine cell subclasses. Tenm4:mCitrine BAC transgenesis was less efficient and exhibited low fluorescence levels so a detailed morphological classification of tenm4 expressing cells could not be established. Tenm3, on the other hand, has been shown to be expressed by RGCs, amacrine cells and also tectal neurons in zebrafish embryos via *in situ* hybridization studies (Antinucci et al., 2013), while a BAC transgenic line with Gal4FF under the transcriptional control of regulatory elements upstream and downstream of the tenm3 start codon, Tg(tenm3:Gal4), was combined with a Tg(UAS:tagRFP-CAAX) responder line and labeled subsets of amacrine and tectal cells (Antinucci et al., 2016). The tenm3-positive amacrine cells consistently stratified their neurites in three IPL strata but did not stratify correctly in tenm3 knockout mutants (Antinucci et al., 2016). Indeed, tenm3 expression is critical for the proper development of orientation selectivity in the retina (Antinucci et al., 2016; Antinucci and Hindges, 2018). Further studies into the classification of different teneurin positive cells, will allow us to potentially relate distinct RGC and amacrine cell types to visual functionalities based on their morphology and stratification, with BAC transgenesis being a viable method for accomplishing this.

While the functional dimerization of teneurin into covalent, disulphide-linked homodimers mediates many of its physiological effects via homophilic interactions, all four teneurin paralogs may also participate in the formation of heterodimers (Feng et al., 2002). If multiple teneurins are expressed in a single cell type, it would be prudent to suggest that different hetero- and homodimer combinations could be utilized to establish a range of hetero- and homophilic interactions at the cell membrane. Combined with alternative splicing events and heterophilic interactions with other cell adhesion molecules, the teneurins may form part of a wider molecular code that functions to increase the diversity of cellular interactions from a limited set of genes. In this fashion, different cell adhesion molecules that are specific for a certain cell type may interact in a combinatorial fashion to drive synaptic specificity.

Much of the focus on teneurin functionality in circuit assembly has been through studying tenm3 (Antinucci et al., 2016; Berns et al., 2018). The functional involvement of the other three paralogs, if any, is less well established and more research is needed to shed light on whether these other teneurins may also act in a similar way to regulate the precise connectivity of the nervous system during development. With genetic variations in the human teneurin gene loci implicated as a significant susceptibility factor in many neurological disabilities such as bipolar disorder (Sklar et al., 2011), intellectual disability (Tucker and Chiquet-Ehrismann, 2006) and autism spectrum disorders (Nava et al., 2012), the involvement of the teneurins in conferring precise neural circuitry is paramount. Future studies will build up on the expression data we have presented here, relating the sites of expression to teneurin functionality and synaptic connectivity. Furthering our understanding of this enigmatic process will help bring us a step closer to understanding how the intricate connections of the nervous system are established.

## Data Availability

All datasets generated for this study are included in the manuscript and/or the Supplementary Files.

## Ethics Statement

This work was approved by the local Animal Welfare and Ethical Review Body (King’s College London), and was carried out in accordance with the Animals (Scientific Procedures) Act 1986, under license from the United Kingdom Home Office to RH.

## Author Contributions

RH designed the study. KT, MR-C, and PA carried out the experiments. KT, MR-C, PA, and AC analyzed the data. AC and RH wrote the manuscript with input from the other authors.

## Conflict of Interest Statement

The authors declare that the research was conducted in the absence of any commercial or financial relationships that could be construed as a potential conflict of interest.
