# Supplementary material for: Expression and Roles of Teneurins in Zebrafish
Source: Front Neurosci. 2019 Mar 12;13:158. doi: 10.3389/fnins.2019.00158 (PMC6423166; doi:10.3389/fnins.2019.00158)
Supplement: Supplementary file 1 [file Data_Sheet_1.docx]

**Supplementary Material**


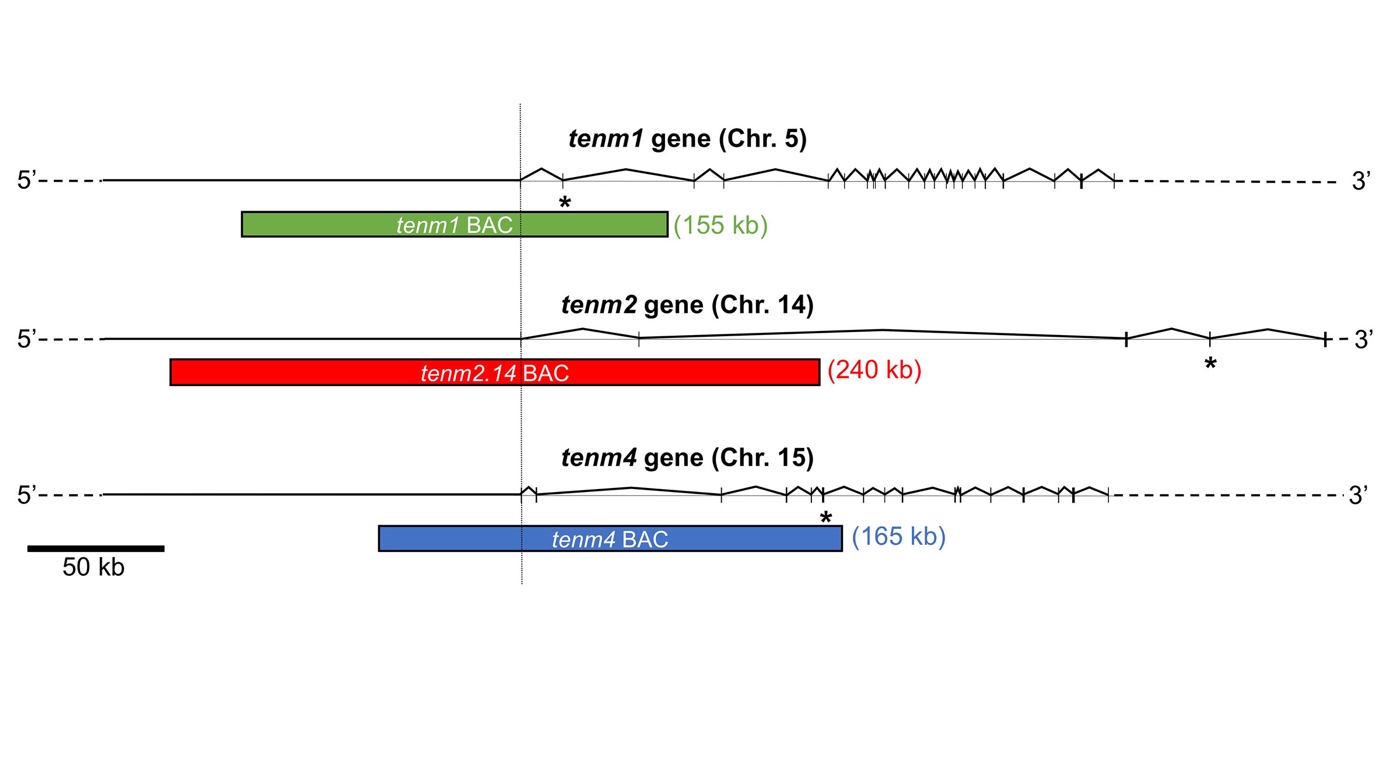


**Supplementary Figure 1: Genome coverage for tenm1, tenm2.14 and tenm4 BAC clones.** Exons are depicted as solid vertical bars and introns as solid lines. Exons, introns and BACs are drawn to scale with the BAC approximate size shown in brackets. Ensembl Gene ID ENSDARG00000003403 for tenm1, ENSDARG00000037122 for tenm2.14 and ENSDARG00000034264 for tenm4. The asterisk indicates the exon with the start of the transmembrane domain.
